# Probiotic treatment differentially affects the behavioral and electrophysiological aspects in ethanol exposed animals

**DOI:** 10.22038/ijbms.2020.41685.9846

**Published:** 2020-06

**Authors:** Amir Hosein Hadidi Zavareh, Ramin Haji Khani, Bahareh Pakpour, Masoud Soheili, Mahmoud Salami

**Affiliations:** 1 Department of Biology, North Tehran Branch, Islamic Azad University, Tehran, Iran; 2 Department of Biology, Faculty of Sciences Central Tehran Branch, Islamic Azad University, Tehran, Iran; 3 Physiology Research Center, Institute for Basic Sciences, Kashan University of medical sciences, Kashan, Iran

**Keywords:** Ethanol, LTP, Memory, Probiotics, Rat

## Abstract

**Objective(s):**

Harmful effects of alcohol on brain function including cognitive phenomena are well known. Damage to gut microbiota is linked to neurological disorders. Evidence indicates that intestinal flora can be strengthened by probiotic bacteria. In this study, we evaluated the effect of probiotics administration on LTP induction in rats receiving ethanol.

**Materials and Methods:**

To assess if probiotic treatment influences toxic effect of ethanol, vehicle (CON) and probiotic treated (CON+PRO) control rats, and chronic ethanol (CE) exposed and CE probiotic treated (CE+PRO) animals were entered into the experiments. Shuttle box test and *in vivo* electrophysiological recordings were accomplished to evaluate memory and hippocampal baseline filed excitatory postsynaptic potentials (fEPSPs) and long term potentiation (LTP), respectively.

**Results:**

Ethanol impaired memory in the CE rats. It also diminished the slope size of fEPSPs and prevented LTP induction. While the probiotic supplementation improved memory in the CE+PRO rats, it did not influence synaptic transmission in these animals.

**Conclusion:**

Conclusively, behavioral but not electrophysiological aspect of cognition is sensitive to probiotic treatment in the ethanol exposed animals.

## Introduction

Numerous preclinical evidences have indicated that gut microbiota, known as a silent organ of the human body, has a favorable effect on cognitive functions (1). The intestinal flora contains about 150 folds more genes than the human genome (2). Importance of normal gut microbiota in NMDA-dependent hippocampal memory has been indicated (3). 

Probiotics, known as beneficial live microorganisms, are considered in human health. Evidence indicates that probiotic bacteria consumption improves defective gut microbiota (4). Our previous study show that supporting the intestinal flora is promising in treatment of neurological disorders (5). It is revealed that some probiotic bacteria including *Lactobacilli* and *Bifidibacterium* reduce expression of GABA_A_ receptors in the hippocampus (6). Also, it is shown that expression of NMDA receptors is increased under probiotic influence (3). Conversion of GABA to glutamate by probiotic bacteria has also been confirmed (7, 8). 

Long term potentiation (LTP) is a candidate mechanism involved in memory formation in the hippocampus (9). The NMDA receptors are the main excitatory receptors involved in induction of LTP. Conversely, activity of the GABA receptors negatively affects synaptic plasticity (10, 11). 

Progressive disruption in learning and memory is related to long term exposure to ethanol (12). Chronic ethanol administration causes cognitive dysfunction from mild impairment to severe anterograde amnesia (13). It has several side effects such as neurotoxicity, neuropathological alterations, and abnormal morphology. It is found that alcohol causes cognitive deficit as well as modifying emotional behaviors, especially in human adolescents (14). The impairment degree is related to the time and dose of ethanol exposure and genetic susceptibility as well (15). The number and subunit composition of the NMDA receptors determine their sensitivity to ethanol exposure (16). Direct effect of alcohol on NMDA receptors disrupts Ca^2+^ influx, a critical ion underlying the synaptic transmission (17, 18). It is reported that alcohol treatment changes capacity of synaptic plasticity and inhibits LTP *in vivo* and *in vitro* (19, 20). Due to improving effect of probiotics on learning and memory, the purpose of this study is to evaluate effect of a probiotic supplement on behavioral and electrophysiological features of rats chronically exposed to ethanol. 

## Materials and Methods

***Animals***

For this study, 28 adult male Wistar rats weighing 150–180 g were provided by the Experimental Animal Breeding Center of Kashan University of Medical Sciences (Kashan, Iran). Experiments were carried out according to the Guidelines of Ethical Committee of Islamic Azad University, Tehran Medical Branch (IR.IAU.TMU.REC.1398.105-1398/06/11). Rats were kept under constant 12-12 hr light-dark cycle at 24± 2 ^°^C, humidity of 60% and access to food and water *ad libitum*. 

***Experimental groups***

The animals randomly were assigned to the 4 following groups: two control groups received either drinking water (CON, n=8) or probiotic supplementation (CON+PRO, n=7). Another two groups received either ethanol (CE, n=5) or ethanol and probiotics (CE+PRO, n=8).

***Probiotics and ethanol administration***

The probiotic supplement consisted of *Lactobacillus acidophilus, Bifidobacterium bifidum, and Bifidobacterium longum* (Zist Takhmir Company-Tehran, I.R. Iran). The probiotic bacteria were capsulated. Each capsule contained 500 milligrams of bacteria with a total CFU of 1×10^9^. The probiotic supplementation was administered through intragastric gavage lasting for 52 days (21). Ethanol was dissolved in drinking water (20% V/V) (22) and administered chronically for 52 days. After that, behavioral training was done for 2 days and followed by electrophysiological recording.

***Behavioral testing***

The shuttle box apparatus consisted of two light and dark segments with equal size (20 × 80 × 20 cm) that were separated by a guillotine door. This passive avoidance test includes training and retrieval or memory phases. In this study the method was performed as previously described (23). Briefly, in the training phase, each rat was placed in the light chamber for 5 sec and then the door was opened and the animals were allowed to move freely into the dark chamber. Upon entry, the door was closed and each rat was given an electrical shock (1.5 mA) in 3 sec. Animals remained in the dark chamber for 20 sec and then were returned to the home cage. In the memory phase (24 hr after training), the rats were placed in the light compartment again and the delay in entering the dark compartment was recorded. More delay to enter the dark chamber indicates successful passive avoidance response. The total time of the test was 300 sec.

***Electrophysiology***

*Animal preparation and electrode implantation*

After behavioral training, animals were anesthetized by urethane (1.5 g/kg, IP) and fixed in a stereotaxic apparatus (WPI’s Precision Stereotaxic Instrument) for extracellular recordings. Based on rat brain atlas (24), two holes were drilled above the skull for stimulator (1 mm diameter, 3.4 mm posterior to bregma, 2.5 mm lateral to the midline, placed in the CA3) and recorder (1 mm diameter, 4.2 mm posterior to bregma, 3.8 mm lateral to the midline, placed in the CA1) electrodes. Teflon-coated stainless steel wire with outside diameter of 0.008 inch (A-M systems, USA) was used and exposed only at the tip (tip separation approximately 0.10 mm).

*Recording procedure*

In response to applied pulses to the Schaffer collaterals pathway, fEPSPs were recorded in the CA1 area of the hippocampus. When the responses were stable, stimulus intensity was determined by the input/output curve. For all steps, the stimulation intensity was adjusted to a level that evoked a 60% of maximum fEPSPs. The baseline fEPSPs were recorded for 30 min with 30 sec intervals. The recording fEPSPs were amplified by a preamplifier (Electromadule, WSI, IR Iran), filtered at 1–3000 Hz, digitized (10 points/ms), and stored for offline analysis using the Potentalize software package (WSI, IR Iran). Then, using a high frequency stimulation (HFS) of 100 Hz (10 bursts of 10 stimuli, 0.1 mili-sec stimulus duration and 2 sec inter-burst interval), LTP was induced. Following the tetanus, responses to the test pulses were collected continuously for 60 min.

***Data analysis***

One-way ANOVA followed by Tukey’s post-test was applied on the data pooled from both behavioral and electrophysiological experiments. In the passive avoidance test, the delay to enter the dark compartment was evaluated. The slope of fEPSPs was considered for electrophysiological assessments. The pre and post-tetanic changes in the slope (mV/ms) were compared to evaluate occurrence of LTP in the post-HFS responses. To normalize all data, the pre-tetanus slope of fEPSPs was taken as 100% (as baseline) and slope of the post-tetanus fEPSPs were compared with it. The calculation was as: post-tetanus value – pre-tetanus mean / pre-tetanus mean × 100 + 100.

All data are presented as mean±SEM and the probability levels were considered statistically significant if *P*-value was less than 0.05.

## Results

***Cognitive performances***

**Figure 1:**
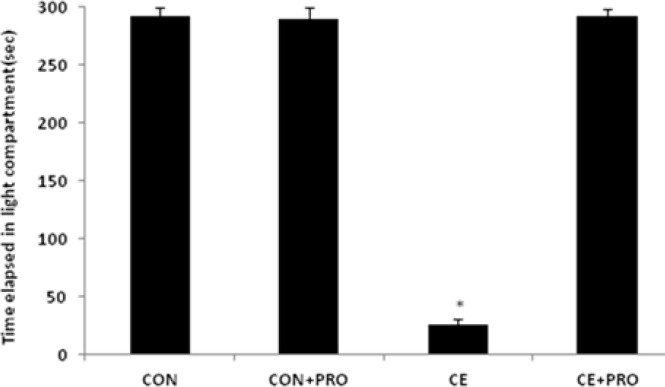
Comparison of the secondary latency time in the passive avoidance test between groups. The data indicated that CE rats spent less time in the light compartment compared to other groups (*P<0.0001 vs CON group)

Ethanol deteriorated the memorizing ability where the latency times to enter the dark compartment were 25.86±7.22 sec and 292.80±7.20 sec in the CE and CON groups, respectively (*P*<0.0001). Probiotic treatment restored the implicit cognition such that the CE+PRO group stayed 290.00±10.00 sec in the light chamber (*P*<0.0001, versus CE group). Figure 1 showed that the supplementation did not effectively underlie the memory capability in the normal reared animals where the latency value in the CON+PRO group was almost the same as in the CON ones (291.67±5.43). 

***Electrophysiological recordings***

**Figure 2:**
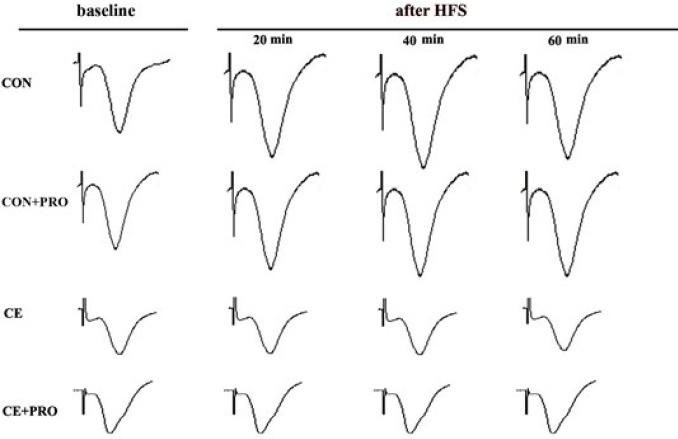
Representative spikes of pre- and post-tetanus slopes of hippocampal fEPSPs over the first, second, and third 20 min of recording. Each trace represents an average of ten consecutive records

The pre-HFS baseline fEPSPs in CA1 of the hippocampus were recorded in response to stimulation of the Schaffer’s collaterals. Post-HFS responses were recorded for 60 min to assess the plasticity level of CA3-CA1 pathway. General ANOVA indicated a significance difference between the testing groups (F_3, 1676_= 29.08, *P*<0.0001). Figure 2 represents spikes of pre- and post-tetanus fEPSPs over the first, second, and third 20 min of recording.

***The baseline recordings in the CA1 area of the hippocampus***

**Figure 3:**
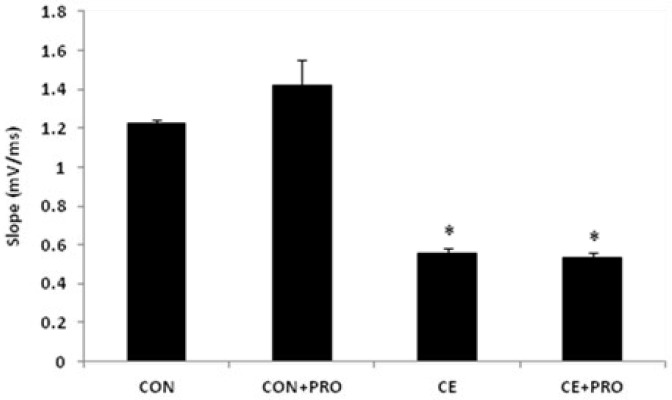
Mean amplitude of the baseline fEPSPs recorded from the CA1 area of the hippocampus in response to the Schaffer's collaterals. The baseline fEPSPs have been declined in CE and CE+PRO animals compared to the CON group (*P<0.0001) while the CON+PRO rats have the same amplitude as CON (*P*=0.12)

The electrophysiological recordings showed that ethanol significantly suppresses the baseline responses (Figure 3). While the mean slop size of fEPSPs in the CON group was 1.22±0.02 mV/ms, it decreased by about half (0.55±0.03 mV/ms) in the alcohol treated CE animals (*P*<0.0001). Analogous magnitude of fEPSPs was achieved in the probiotic-administered CE+PRO rats and their CE counterparts (0.53±0.23 and 0.55±0.26 mV/ms, respectively). We observed a negligible positive effect of probiotic treatment in the baseline responses taken from the CON+PRO group (1.41 ±0.13 mV/ms; *P*=0.12 vs CON group). 

***Induction of LTP in the fEPSPs of the CA1 area of the hippocampus***

**Figure 4:**
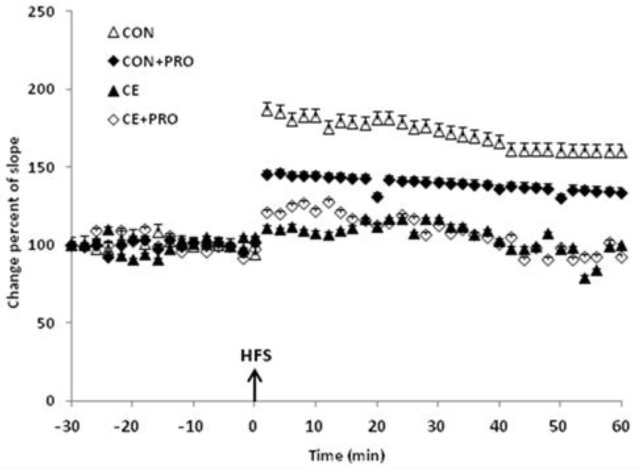
The percent change of post-tetanus fEPSPs. The tetanic stimulation considerably triggered LTP in post-tetanus responses in CON and CON+PRO rats (*P*<0.0001 compared to baseline). On the other hand, it failed to elicit a maintained potentiation in the fEPSPs in CE and CE+PRO animals. Arrow indicates the time of application of the high frequency stimulation (HFS). Each point indicates data average obtained during 2 min

Tetanization induced a considerable LTP in fEPSPs recorded in the CON group. Post-tetanus recordings indicated a steady enhancement over experiment (*P*<0.0001). The probiotic treated CON+PRO group also showed a post-tetanic potentiation (*P*<0.0001). Administration of chronic alcohol efficiently prevented occurrence of LTP so that no considerable change was observable in the post-HFS compared to the baseline responses recorded in the CE rats. Post-tetanus fEPSPs in the probiotic treated CE+PRO group did not significantly vary compared to their CE counterparts either (Figure 4). 

## Discussion

Ethanol is a common antibacterial and antifungal substance that is being abused in many societies. Since alcohol consumption causes brain damage and behavioral changes, hence, early detection of health problems induced by ethanol is important (25, 26). Findings of the present study showed that chronic ethanol administration affects memory in animals. The data taken from shuttle box revealed that a 52-day ethanol treatment led the rats to show less latency time in the light chamber, indicating memory defect in the alcohol treated animals. 

LTP is a nominated experimental technique to evaluate synaptic plasticity in neuronal circuits (27). Induction of LTP in Shaffer-collateral pathway depends on NMDA receptors. Indeed, NMDA receptors have an important function in excitatory synaptic transmission in the hippocampus. We found that chronic ethanol treatment destroys induction of LTP in the hippocampus. These data are in compliance with previous studies that chronic ethanol administration contradicts memory formation (28, 29). 

The cholinergic system is also suppressed in the hippocampus of alcohol treated subjects. This, in turn, causes a reversible cognitive deficit in animal models (30, 31). Histologically, administration of alcohol for a long period of time leads to non-uniform morphological and neurochemical alterations in the central nervous system, particularly in the hippocampus (32, 33). Since the hippocampus is involved in the memory process, chronic alcohol intake leads to memory and cognition impairment. Consistently, it has been proven in animal studies that alcohol induced impairment of spatial and non-spatial working memory as well as object recognition tasks (34). 

The intestinal microbiota flora plays an important role in the host health. It affects numerous brain functions including behavior, CNS development, learning, and memory (35, 36). Hence, current strategy in food industries supports the gut microbiota (37). In this study, it was revealed that probiotic bacteria could reverse deteriorating effect of alcohol on memory in shuttle box strategy. Research in animal models indicates that administration of a probiotic mixture favorably improved impaired learning and memory (38, 39). There are infrequent documents which have evaluated irritating effect of probiotics on brain neurochemistry and its synaptic plasticity. The probiotics, or as a whole intestinal flora, can impact brain activity through prompting of production of some neuromodulators or neurotransmitters including BDNF, GABA, serotonin, dopamine, norepinephrine, and acetylcholine (40-42). Germ free animal study by McVey Neufeld *et al*. demonstrated that commensal gut flora modulates normal excitability of gut sensory neurons (43). 

Two separate investigations proved that gut microbiota is able to affect myelination and neurogenesis. It is also confirmed by our previous animal study that administration of probiotic effectively restored diminished hippocampal LTP (44, 45). Spatial and associative memory improvement was observed in rats treated by *Enterococcus faecium *(46). Our experimental (21) and clinical (47) studies also indicate that probiotic supplementation may positively affect cognitive considerations. From these considerations and findings in other animal models of impaired memory, it seems that probiotic treatment positively affects the deteriorated cognitive function in the CE animals.

From the view point of electrophysiology, however, probiotic consumption could not influence LTP suppression in CE rats. Evidence indicates that the effect of probiotic bacteria on plasticity of synaptic transmission is scant. Favorable effects of probiotics on hippocampal LTP has been documented in a few studies. We showed that probiotics restore impaired LTP in an animal model of diabetes (21). Distrutti *et al*. showed that probiotic consumption successfully restored impaired synaptic plasticity in aged rats by a negligible effect on microglial activation markers and over expression of BDNF and synapsin (48). A study found that probiotics treatment leads to a robust LTP in a model of middle-aged rats (46). 

## Conclusion

Taken together, it seems despite beneficial effect of probiotics on behavioral aspects of cognition, however, intervention cannot influence direct synaptic activity. Further investigations are required to clarify which features of synaptic transmission are sensitive to ethanol administration and probiotic treatment.
